# Resistance mutations of NS3 and NS5b in treatment-naïve patients infected with hepatitis C virus in Santa Catarina and Rio Grande do Sul states, Brazil

**DOI:** 10.1590/1678-4685-GMB-2018-0237

**Published:** 2020-02-17

**Authors:** Elisabete Andrade, Daniele Rocha, Marcela Fontana-Maurell, Elaine Costa, Marisa Ribeiro, Daniela Tupy de Godoy, Antonio G.P. Ferreira, Amilcar Tanuri, Rodrigo Brindeiro, Patrícia Alvarez

**Affiliations:** 1 Fundação Oswaldo Cruz/Fiocruz, Instituto de Tecnologia em Imunobiológicos Bio-Manguinhos, Rio de Janeiro, RJ, Brazil.; 2 Universidade Federal do Rio de Janeiro (UFRJ), Rio de Janeiro, RJ, Brazil.

**Keywords:** Direct-acting antivirals, resistance-associated substitutions, blood donors, NS3, NS5b

## Abstract

Hepatitis C virus (HCV) infection is a worldwide health problem. Nowadays, direct-acting antiviral agents (DAAs) are the main treatment for HCV; however, the high level of virus variability leads to the development of resistance-associated variants (RAVs). Thus, assessing RAVs in infected patients is important for monitoring treatment efficacy. The aim of our study was to investigate the presence of naturally occurring resistance mutations in HCV NS3 and NS5 regions in treatment-naïve patients. Ninety-six anti-HCV positive serum samples from blood donors at the Center of Hematology and Hemotherapy of Santa Catarina State (HEMOSC) were collected retrospectively in 2013 and evaluated in this study. HCV 1a (37.9%), 1b (25.3%), and 3a (36.8%) subtypes were found. The frequency of patients with RAVs in our study was 6.9%. The HCV NS5b sequencing reveled 1 sample with L320F mutation and 4 samples with the C316N/R polymorphism. The analysis of the NS3 region revealed the D168A/G/T (3.45%), S122G (1.15%), and V55A (2.3%) mutations. All samples from genotype 3a (36.8%) presented the V170 I/V non-synonymous mutation. In conclusion, we have shown that mutations in NS3 and NS5b genes are present in Brazilian isolates from therapy-naïve HCV patients.

## Introduction

Hepatitis C virus (HCV) infection is a worldwide health problem. According to the Global Hepatitis Report, from the World Health Organization (WHO), approximately 71 million people have chronic HCV infection, and nearly 399,000 people die each year, mostly due to cirrhosis or hepatocellular carcinoma (WHO, 2017). HCV has a high genetic heterogeneity and is classified into seven genotypes (1 to 7) and 67 subtypes (Smith *et al.*, 2014). The genotype distribution depends on geographical location and risk groups (Cantaloube *et al.*, 2005). Genotype 1 is the most frequent in Brazil, followed by genotypes 3 and 2 (Campiotto *et al.*, 2005; Lampe *et al.*, 2013).

There is no vaccine available for preventing HCV infections. The main antiviral treatment until 2011, was PEGylated interferon-alfa (αPeg-IFN) alone or in combination with ribavirin, leading to a sustained virological response (SVR) in 50% of treated patients, depending on the virus genotype causing the HCV infection (Peres-da-Silva *et al.*, 2012; Paolucci *et al.*, 2013; Gross *et al.*, 2018). Nowadays, direct-acting antiviral agents (DAAs) have been approved for HCV infection treatment, with an average SVR above 95%, at least for genotypes 1 and 4 (Leuw and Stephan, 2018). In Brazil, DAAs were incorporated by the Ministry of Health for the treatment of hepatitis C under the Unified Health System (SUS) since 2015 (Ministério da Saúde, 2018). Unfortunately, there is little data about the efficacy of DAAs in Brazil, with some information found in the study by Sette Jr *et al.* (2017).

The primary targets of DAAs are nonstructural proteins essentials for HCV replication, which include the NS3 protease, NS5B polymerase, and NS5A protein (Paolucci *et al.*, 2013; Lontok *et al.*, 2015). However, a challenge in HCV treatment is the emergence of viral resistance mutations that reduces susceptibility of the virus to DAA therapies (Hoffmann *et al.*, 2015; Gededzha *et al.*, 2017). The development of resistance-associated variants (RAVs) is due to the high level of virus variability, from the combination of the virus’ high replication rate, low RNA polymerase fidelity rate, and selective pressure for drug or immunomediated treatment (Peres-da-Silva *et al.*, 2012; Paolucci *et al.*, 2013; Gededzha *et al.*, 2017).

The presence of RAVs in patients not yet under treatment has been reported previously in different countries (Peres-da Silva *et al.*, 2010; Paolucci *et al.*, 2013; Zeminian *et al.*, 2013; Gededzha *et al.*, 2017). In addition, a systematic review regarding HCV resistance-associated substitutions and their clinical relevance was published recently (Sorbo *et al.*, 2018). Therefore, assessing RAVs in infected patients is important for monitoring the efficacy of therapy (Loggi *et al.*, 2017) and the epidemiology of HCV in Brazil. Thus, the aim of our study was to investigate the presence of naturally occurring resistance mutations in HCV NS3 and NS5 regions in treatment-naïve patients.

## Materials and Methods

The Center of Hematology and Hemotherapy of Santa Catarina State (HEMOSC) is currently responsible for the nucleic acid testing for HIV, HCV, and HBV in samples from blood donors from Santa Catarina and Rio Grande do Sul states. Annually, HEMOSC receives around 300 thousand blood donations. A total of 96 samples that were positive for HCV in 2013 were used for this study, retrospectively.

HCV RNA was extracted from plasma previously conserved at -80 °C using a molecular biology workstation (BioRobot MDx, Qiagen), with the Qiamp one-for-all nucleic acid kit (Qiagen), according to the manufacturer’s instructions. Plasma HCV RNA was quantified using COBAS/Taqman HCV Test v2.0 (Roche).

Genotyping/subtyping was performed by amplifying and sequencing a 339-bp amplicon of the NS5b region, according to Cantaloube *et al.* (2005). The nucleotide sequences obtained were analyzed in the Geno2pheno _[HCV]_ (Kalaghatgi *et al.*, 2016) for genotypes and subtypes, and possible resistance against licensed DAAs.

The amplification of the entire NS3 region of the HCV genome, followed by a second PCR was performed as described previously (Peres-da-Silva *et al.*, 2010), using primers specific to subtypes 1a, 1b, and 3a. The nucleotide sequences obtained from each subtype were analyzed for drug resistance in the Geno2pheno _[HCV]_ (Kalaghatgi *et al.*, 2016).

The statistical program SPSS (IBM SPSS Statistics Base 22.0) was used. Multivariate analysis of variance (ANOVA) was applied to compare means of continuous variables with normal distribution *(p*<0.05).

## Results and Discussion

From the 96 HCV-positive samples collected, nine did not have enough material to perform the assays and were excluded from the analysis. Eighty-seven samples were used for genotyping and analysis of NS3 and NS5b regions.

From the 87 samples evaluated, 33 (37.9%) were of genotype 1a, 22 (25.3%) were of genotype 1b, and 32 (36.8%) were of genotype 3a. Genotype 1 (1a plus 1b) was the most frequent, followed by genotype 3, a result that is in agreement with what was previously reported in Brazil (Campiotto *et al.*, 2005; Lampe *et al.*, 2013; Nishiya *et al.*, 2014). We did not find genotypes 2, 4, and 5, known to be less frequent in Brazil. Sixty-three samples were successfully geno- and subtyped by the NS3 and NS5b region, and there was no disagreement between the HCV genotypes in both regions. Twenty-four samples had no amplification of the NS5b region, even with an alternative protocol (Sandres-Sauné *et al.*, 2003), and were thus genotyped according to Peres-da-Silva (2010) protocol (NS3 region). This amplification divergence has already been discussed in Larrat *et al.* (2013), who reported a failure of some quantitative RT-PCR assays to detect or amplify correctly the NS5b region in some strains of HCV, even when using three sets of primers covering two different regions. This could be explained by the great variety of viruses, the use of primers not suitable for these peculiar strains, or by a mixed infection in the plasma sample.

The mean viral loads were 5.31 log IU/mL for genotype 1a, 5.18 log IU/mL for 1b, and 5.38 log IU/mL for 3a. There was no difference in viral load between the genotypes (*p*=0.6). The detected HCV genotypes and viral loads are both important predictors for therapeutic outcomes. It has been reported that patients infected with genotype 1 are more likely to have higher viral loads than those infected with genotype 2 and 3 (Scott *et al.*, 2007; Soriano *et al.*, 2008; Nishiya *et al.*, 2014). In contrast to our results, in a study carried out with blood donors from São Paulo, the viral load from genotype 3a (5.22 log10 IU/mL) had a lower log mean than genotype 1a (5.99 log10 IU/ mL) (*p*=0.0002) and genotype 1b (6.35 log10 IU/mL) (Nishiya *et al.*, 2014), in agreement with a previous report.

**Figure 1:**
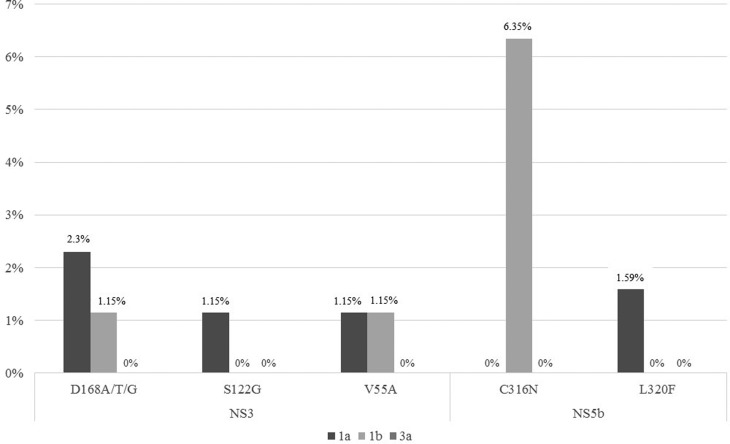
Frequency of specific NS3 and NS5b resistance-associated variants found in this study by HCV subtype.

The frequency of patients with RAVs in our study (6.9%) was intermediate when compared with other Brazilian studies among HCV chronic carriers not treated with protease inhibitors (3.2% - 18.9%) (Hoffmann *et al.*, 2013; Nishiya *et al.*, 2014). Figure 1 present the frequency of specific NS3 and NS5b resistance-associated variants found in this study by HCV subtype.

The HCV NS5b sequencing from 63 samples was analyzed. The L320F mutation was present in only one sample (1.59%) of genotype 1a. L320F is known to confer low resistance to sofosbuvir and sofosbuvir associated with mericitabine (Paolucci *et al.*, 2013), which are associated to treatment failure in clinical trials (Constantino *et al.*, 2015). In a previous study, L320F single mutation had no significant impact on the 50% effective concentration (EC50) and EC90 values for mericitabine (≤2.7 fold) (Tong *et al.*, 2014). To our knowledge, this is the first time that mutation L320F is reported as naturally occurring in DAA treatment-naïve patients and it should be monitored due to treatment failures reported previously in clinical trials.

The polymorphism C316N/R was present in 4 samples (6.35%) of genotype 1b. C316N is reported to confer low level of resistance to sofosbuvir (Paolucci *et al.*, 2013, Lontok *et al.*, 2015). C316N mutation has been associated with a 10-fold increase in EC50 to a new experimental non-nucleoside drug, HCV796 (Castilho *et al.*, 2011). Previous studies have found variable prevalence of C316N in Brazil, from 3.85% (Peres-da-Silva *et al.*, 2017) to 11.6% (Castillho *et al.*, 2011), 16.3% (Noble *et al.*, 2017), and 24% (Castilho *et al.*, 2011), and higher prevalence in North America (16.81%), Europe (7.47%), and Asia (49.71%) (Peres-da-Silva *et al.*, 2017). The higher prevalence of mutations in genotype 1b has been reported previously and it was due to the presence of C316N (Paolucci *et al.*, 2013; Peres-da-Silva *et al.*, 2017).

Although it was not a goal of our study, we also observed the presence of D244N, Q309R, and A333E mutations conferring resistance to ribavirin and interferon in 42 samples (57.14%). Twenty samples (26.98%) presented Q309R, and three (1.58%) A333E. Seventeen were Q309R and D244N, two were Q309R and A333E, and two were triple positive. In a previous work, the most frequent mutation observed in Brazil was Q309R, present in all HCV subtypes (Castilho *et al.*, 2011); in our study, it was present in 38 samples. No double mutations in the NS5b region conferring resistance to DAAs was observed in our samples. The emergence of double or triple-sites RAVs in the clinics is threatening the effectiveness of anti-HCV therapies, as published previously (Gane *et al.*, 2016).

The analysis of the NS3 region revealed the mutations D168A/G/T (3.45%, 3/87), S122G (1.15%, 1/87), and V55A (2.3%, 2/87) that confer resistance to asunaprevir, boceprevir, grazoprevir, simeprevir, and paritaprevir (Zeminian *et al.*, 2013; Lontok *et al.*, 2015; Sorbo *et al.*, 2018). V55A was observed at a higher frequencies in previous works, at 4.1% (Moreira *et al.*, 2018) and 6% (Nishiya *et al.*, 2014), from DAA naïve patients and blood donors, respectively, in São Paulo, and at 6% in Europe (Bartels *et al.*, 2013). The V55A variant has been shown to confer 6.9-fold increase in EC50 to boceprevir (Vermehren *et al.*, 2012). S122G was found in a higher frequency in Spain (6.23%) and China (85.48%) (Li *et al.*, 2017). An *in vitro* study has shown that S122G did not reduce susceptibility to simeprevir (Izquierdo *et al.*, 2014). However, another study showed that S122G reduced the susceptibility by 0.5-fold (Lenz *et al.*, 2010). In São Paulo, the D168G mutation was found in one of the 125 HCV infected blood donors’ samples (Nishiya *et al.*, 2014). In a transient susceptibility assay, D168G conferred low- to moderate-level asunaprevir resistance (5- to 21-fold) for HCV genotype 1a. For genotype 1b, a higher level of asunaprevir-associated resistance was observed ranging from 170- to 400-fold relative to wild-type control. (McPhee *et al.*, 2012).

No mutations were found that confer resistance to glecaprevir and voxilaprevir, drugs known to present a high barrier to resistance (Sorbo *et al.*, 2018). However, this study found samples with mutations that decrease the susceptibility of HCV to these drugs, which reinforces the importance of monitoring HCV RAVs.

Samples from genotype 3a presented no mutations that confer or diminish resistance to glecaprevir and voxilaprevir, drugs recommended for treatment of patients infected with this genotype. This means that the standard protocol for treatment of patients with genotype 3 should be effective in Santa Catarina and Rio Grande do Sul. However, we found the non-synonymous mutation V170 I/V in all 32 samples of this genotype. In agreement with our results, Peres-da-Silva (2010) found that 100% (32/32) of the HCV 3a sequences contained the V170I substitution. Few data is available on effects of V170I substitution. The conservative substitution at this site was detected in up to 45% of patients infected with HCV genotype 1 (López-Labrador *et al.*, 2008)

A different pattern of resistance associated with NS3 protease domain in therapy-naïve patients was previously reported in Brazil. V36L mutation was found in genotype 1a at a frequency of 5.6%, in 1b at 100% (Peres-da-Silva *et al.*, 2010), and in genotypes 2, 3, 4, and 5 V36L mutation was found as a genetic signature with frequency of 99% (Vidal *et al.*, 2016); in another work, V36L was found at a frequency of 4% in genotype 1a (Nishiya *et al.*, 2014). T54S mutations were found in 4.1% of genotype 1a (Peres-da-Silva *et al.*, 2010) and 100% in genotype 2 (Vidal *et al.*, 2016). The samples investigated by our study presented none of these mutations. The Q80K, a common mutation in the USA (40%) (Bartels *et al.*, 2013) that confers resistance to simeprevir, was not found in our study, but has previously been reported at prevalence ranging from 0.4% to 2.7% in Brazil (Nishiya *et al.*, 2014; Vidal *et al.*, 2015; Moreira *et al.*, 2018). There is a strong geographic correlation regarding the frequency of the Q80K substitution (Moreira *et al.*, 2018), and for this reason, studies from different geographic regions are of great importance, especially in a large country as Brazil.

Of all the samples evaluated, only one sample of genotype 1b showed mutation in the genes NS3 and NS5b, conferring resistance to sofosbuvir (C316N) and decreased susceptibility to gazoprevir (Y56F). This shows the importance of studying both NS3 and NS5 proteins when evaluating or choosing the therapy strategy for HCV-positive patients. Patients carrying combinations of resistance mutations are of particular interest, since they may increase the possibility of failure in the treatment with DAAs.

The frequency of resistance mutations and genotypes was twice as high among patients with subtype 1a compared to those with subtype 1b. A similar result was found in a study with blood donor’s samples from São Paulo (Nishiya *et al.*, 2014). In addition, a higher frequency of virological failure for subtype 1a compared to 1b has been reported (Pawlotsky *et al.*, 2011).

At last, several polymorphisms not associated with resistance to DAAs were observed in our study (Table 1), and previously reported by others (Constantino *et al.*, 2015). Polymorphisms, prior to therapy, are part of the quasispecies population in infected individuals, and may not alter viral fitness (Peres-da-Silva *et al.*, 2012; Paolucci *et al.*, 2013; Nishiya *et al.*, 2014).

**Table 1:** HCV polymorphic sites distribution according to genotype.

Gene	Position	Polymorphisms^*^	1a	1b	3a
**NS3**	62	R62K	7	3	5
64	I64L/M	7	5	2
86	P86Q/H	5	2	7
89	Q89A/H/P	8	3	3
91	S91A/T	18	11	10
102	S102A/F	3	6	8
140	T140A	22		
147	A/F147G/M/S/T	9	2	8
153	L153I	24	9	9
166	A/S166 A/T/R	4	5	9
170	I/V170I/V/H	3	2	32
176	E/S176K/N	8	9	5
**NS5**	244	D244N			17
254	K254R/S	5	13	22
300	R300Q/S/T	17	17	23
309	Q309R/H	15	1	22
312	T312D/E/S/R	2	2	23
329	V329E/F/G/R/T	14	17	7
332	D332G/N/R	5	12	13
333	A333E/G/P/Q	11	6	23
334	A334G/H/V/Q/W	12	10	17
335	S335E/N/G/Q/T	15	13	23
336	L336A/P	14	12	20
337	R337N/T/	11	12	9

* Some samples had more than one variant.

In conclusion, we have shown that mutations in NS3 and NS5b domains are present in Brazilian isolates from therapy-naïve patients, in this case, blood donors with unknown HCV infection. Monitoring the presence of RAVs is important for predicting the response to antiviral therapy, and regional discernment can help determine local policies for treatment. The results presented here will help ensure therapy strategies that are more successful for HCV-infected patients in Santa Catarina and Rio Grande do Sul states in Brazil.
